# Islands, mainland, and terrestrial fragments: How isolation shapes plant diversity

**DOI:** 10.1002/ece3.3150

**Published:** 2017-07-28

**Authors:** Emi Martín‐Queller, Cécile H. Albert, Pierre‐Jean Dumas, Arne Saatkamp

**Affiliations:** ^1^ Aix Marseille Univ Univ Avignon, CNRS, IRD Institut Méditerranéen de Biodiversité et d'Ecologie (IMBE) Marseille France

**Keywords:** amount of habitat area, biodiversity, dispersal, functional connectivity, graph theory, habitat fragmentation, isolation, matrix permeability, species–area relationships, urbanization

## Abstract

The fragmentation of natural habitats is a major threat for biodiversity. However, the impact and spatial scale of natural isolation mechanisms leading to species loss, compared to anthropogenic fragmentation, are not clear, mainly due to differences between fragments and islands, such as matrix permeability. We studied a 500 km^2^ Mediterranean region in France, including urban habitat fragments, continuous habitat, and continental‐shelf islands. On the basis of 295 floristic relevés, we built species–area relationships to compare isolation in fragments after urbanization, with continuous habitat and continental‐shelf islands. We assumed either no dispersal, infinite dispersal, or estimated intermediate levels of habitat reachability through graph theory. Isolation mechanisms occurred in fragments but with a lower strength than in near‐shore islands, and most importantly affected perennial plants. Annual plants were less affected, probably due to their smaller size and shorter life cycle. Isolation occurred at landscape level in fragments and at patch level in islands. The amount of reachable habitat (accounting for spatial configuration) explained local species richness in both systems, but the amount of habitat (no consideration of spatial configuration) was already a good predictor. These results suggest an important role of habitat amount around fragments in mitigating the isolation effects observed in near‐shore islands, and the importance of carefully considering different functional groups.

## 1 INTRODUCTION

Loss of natural habitat is one of the main threats to terrestrial biodiversity (Bradshaw, Sodhi, & Brook, 2009; Butchart et al., 2010). It most frequently results in habitat fragmentation—a decline in the size of remaining contiguous habitat areas (hereafter “fragments”) and their physical separation within the landscape (hereafter “isolation”)(Forman, 1995)—which is thought to disrupt ecological processes and lead to species extinction (Fahrig, 2003). Over the last decades, research and conservation regarding such remaining habitat fragments embedded in a matrix of human land use have been guided by the Theory of Island Biogeography (TIB, MacArthur & Wilson, 1967). Although it proposes powerful concepts, the TIB has been developed for islands—that is, habitat areas surrounded by water—and recent works have emphasized the differences between “islands” and “fragments,” leading to a plea for the development of a theoretical framework specifically suited to fragments (Fahrig, 2013; Laurance, 2008; Matthews, 2015; Mendenhall, Karp, Meyer, Hadly, & Daily, 2014).

Comparing islands to fragments is of prime interest, as islands offer clear evidence of specific biological processes related to their spatial isolation from any other habitat areas. The TIB predicts that the assemblage of species occupying an island results from a balance between three processes: (1) Extinction rate is expected to be lower on larger islands due to reduced demographic drift in larger populations, (2) increased isolation, here understood as the distance to the nearest possible species pool (e.g., continent, other islands), should lead to reduced immigration rates, and (3) speciation rate is expected to be higher on larger and more isolated islands due to increased environmental heterogeneity and reduced gene flow (Hubbell, 2001; MacArthur & Wilson, 1967; Shaffer, 1981). The dominant processes of species addition when going from smaller to larger islands is expected to alter the rate of species richness increase with island area (MacArthur & Wilson, 1967; Preston, 1962). This leads to a negative interaction between the effects of island size and isolation on species richness: When isolation increases (i.e., lower immigration and higher speciation rates), the rate of “species richness accumulation with island area” increases, as shown by Triantis, Guilhaumon, and Whittaker (2012), who found higher rates of species richness increase with island area for oceanic islands (speciation‐dominated system) than for continental shelf islands (immigration‐extinction dynamics), or for inland islands (low‐dispersal limitation systems). Whether these processes related to isolation generate further species extinctions besides those strictly caused by habitat loss in remnant habitat fragments has important implications for biodiversity conservation (Simberloff & Abele, 1976). The little empirical support for such isolation effects in the case of habitat fragments (Fahrig, 2013; Matthews, 2015; Mendenhall et al., 2014) may be due to the critical differences that exist between habitat fragments and islands. Firstly, the matrix in which fragments are embedded is potentially more permeable to organisms' movement than water, thus strongly influencing how the distance between fragments drives immigration rates (Tischendorf & Fahrig, 2000). Secondly, the—generally recent—history of fragments is fundamentally opposed to the long‐term biogeographical history of islands. While islands are expected to host speciation events, fragments are rather expected to be the place for extinction debts that may lead to artificially high species richness due to species that are not yet locally extinct (Diamond, 1972; Tilman, May, Lehman, & Nowak, 1994). Thirdly, the nature of this more permeable matrix could deeply influence the demography and survival of local populations (Laurance, 2008; Brudvig et al., 2017). In addition, differences in local environmental conditions, such as strong winds or salinity, may lead to different species richness between islands and fragments (Field et al., 2009).

A classical approach to compare fragments and islands is to compare the increase in species richness with the habitat area, the so‐called species–area relationship or SAR (e.g., Halley, Sgardeli, & Triantis, 2014; Triantis et al., 2012). However, Fahrig (2013) recently called for a revision of this approach when studying habitat fragments. She argued that investigating the relationships between species richness and area at patch level (fragment or island) was inappropriate. Considering patches as meaningful ecological entities would mean that their area is a good proxy for the number of interacting individuals, thus being a fundamental driver of the demographic processes and biotic interactions that shape local species assemblages. This assumption fails to consider that (1) individuals from small but functionally well‐connected patches may interact with many more individuals than those actually hosted in the patch, due to high immigration rates, (2) individuals from large patches may actually interact with far fewer individuals than those hosted in the patch, due to dispersal limitation (individuals are too far apart within the patch), (3) individuals from different species may perceive the landscape differently based on their habitat requirements and dispersal abilities (niche segregation, Baguette, Blanchet, Legrand, Stevens, & Turlure, 2013). To overcome these limitations, Fahrig (2013) proposes to replace patch area in the definition of SARs by habitat amount, that is, the area of habitat in a given neighborhood independently of its spatial structure. Jackson and Fahrig (2014) propose that such neighborhoods be defined as a set of spatial extents centered on the plot and ranging from the size of a single territory to a disk with a radius well above the average dispersal distance of the study organisms. Metrics of “reachable habitat,” based on graph theory, could help further disentangle effects of the amount and spatial structure of habitat within these neighborhoods (Pascual‐Hortal & Saura, 2006). In graph theory, landscapes are conceptualized as networks of weighted nodes (habitat patches with different qualities), connected by weighted links (potential movement based on properties of the intervening matrix) (Urban & Keitt, 2001). Reachable habitat accounts for the fact that the potential of individuals to interact within a landscape network is not only constrained by distance but also by their relative positions in space; better connected individuals interact more strongly and individuals connected via stepping stones (indirect connection through another patch) can still interact.

**Figure 1:**
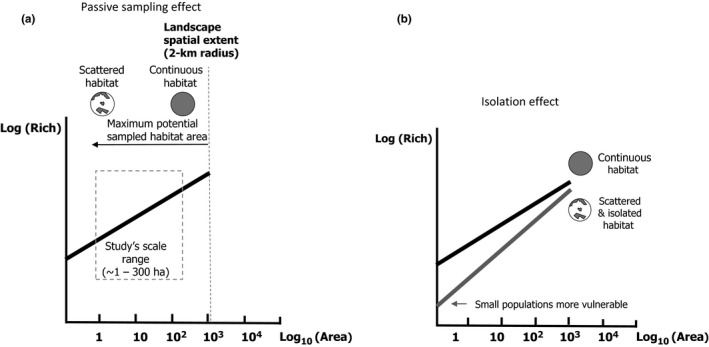
*Passive sampling* versus *isolation Passive sampling* (a) assumes that species richness (“Rich”) measured with a constant sampling effort increases with increasing habitat amount in the neighborhood (“Area”), because more surrounding habitat leads to a higher number of interacting individuals, which necessarily represents more different species for a given species' abundance distribution. Under passive sampling alone, the slope of SARs should be the same, whatever the spatial arrangement of habitat in the surrounding landscape. *Habitat isolation* (b) may play a role with regard to local species richness beyond that of habitat amount. This means that the spatial arrangement of the habitat surrounding a focal patch influences the number of interacting individuals. Isolated habitat undergoes higher extinction, reduced immigration, and higher speciation rates. The SAR slope reflecting an effect of isolation should be steeper than that generated by passive sampling alone. While this pattern has been demonstrated for islands versus continuous habitat, it is less clear in the case of habitat fragments

Fahrig (2013) propose on the basis of these revised SARs—that relate species richness with the total amount of habitat in the “local landscape” or neighborhood of the sample site (or the amount of reachable habitat)—one could compare the slopes of these relationships in a log–log setting for different levels of isolation (continuous habitat vs. habitat fragments), in order to discriminate between two major hypotheses regarding the main drivers of species assemblages: “passive sampling” vs. “isolation effect” (Figure 1). The simplest mechanism that leads to increased species richness with increasing habitat amount is “passive sampling.” It considers species as neutral, that is, it ignores the effects of heterogeneous ecological niches and habitat differences on community dynamics and focuses on the effects of differences in species abundance. It suggests that in smaller areas, fewer individuals are sampled from the regional pool which necessarily represents fewer species for a given species' abundance distribution (Fahrig, 2013; Scheiner et al., 2011; Schoereder & Galbiati, 2004; Turner & Tjørve, 2005). In the absence of isolation effects (the “habitat amount hypothesis,” Fahrig et al. 2013), the slope of SARs should be the same in all cases (Figure 1) and notably should be independent of habitat spatial configuration (Fahrig, 2013; Halley et al., 2014; Watling & Donnelly, 2006). Contrastingly, the SAR slope reflecting an effect of isolation should be steeper than that generated by passive sampling alone (Figure 1; Fahrig, 2013; Halley et al., 2014). The major difficulty in reliably testing these hypotheses and being able to compare different levels of isolation is the necessity of using samples with fixed spatial extents (i.e., equal‐sized plots or quadrats) for which the spatial contexts of community dynamics can be compared (Fahrig, 2013).

Here, we propose to assess the importance of isolation effects on plant assemblages in habitat fragments by comparing SARs for three contrasting scenarios of isolation: continental‐shelf islands, remnant habitat fragments embedded within an urban matrix and a large continuous habitat area. We combine SARs on the basis of species richness in equal‐sized sample sites with the graph‐based amount of reachable habitat in order to test whether species assemblages in habitat fragments result from passive sampling alone, or in combination with isolation effects. Using a dataset of 295 vegetation relevés in the Mediterranean, we ask:

1. Are habitat fragments similar to islands? More precisely, how strongly do patch size and isolation effects interact for fragments, islands, and continuous habitats, when we do not consider connectivity?
2. What would be a relevant neighborhood to model connectivity for plant diversity in patchy landscapes? Notably, is species richness in fragments or islands related to the amount of reachable habitat, as measured by the habitat amount or other graph‐based measures of reachable habitat? What is the relevant spatial scale of isolation processes for habitat fragments and for islands: intra‐patch space, neighboring patches or the surrounding landscape?
3. Do annual and perennial plants differ in their sensitivity to connectivity loss?

Mediterranean coastal ecosystems are highly relevant to address these questions, given their high levels of fragmentation due to a strong historical and current urban pressure (Blondel & Aronson, 1999). We build SARs under realistic scenarios of community size and isolation, based on graph theory and following a neutral approach. Ten scenarios of species dispersal capacity (from 100 m to 1 km) were assessed, and two groups of species with different life forms (annuals vs. perennials) were compared.

## 2 METHODS

### 2.1 Study area

**Figure 2:**
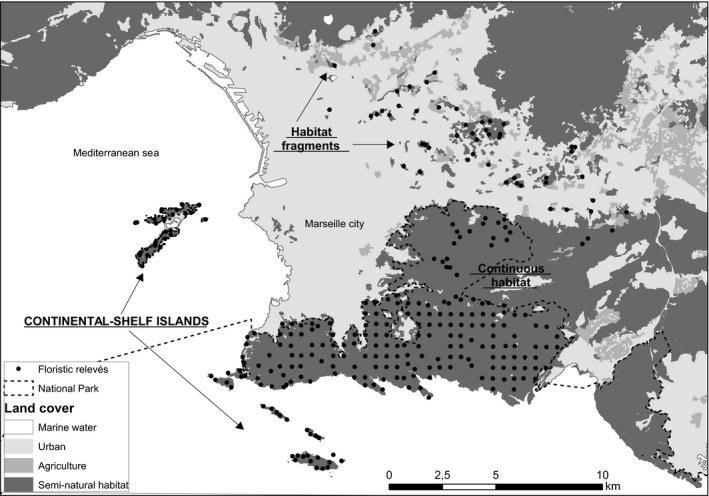
Study area and relevés. Three contrasted scenarios of isolation of natural habitat areas are indicated: continuous habitat, remnant habitat fragments embedded in an urban matrix and continental‐shelf islands

The study area covers an area of 20 × 25 km in Mediterranean France (Figure 2). It includes the city of Marseille, the second largest city in France, and it is surrounded by limestone outcrops that peak at 641 m above sea level. The hills along the southern periphery of Marseille and two limestone archipelagos of continental‐shelf islands acquired the status of National Park (Parc National des Calanques) in 2012, thus limiting the possibility of further urban sprawl in this area. The Marseille islands are near‐shore continental‐shelf islands, they are of continental origin, and were separated from the mainland as recently as ±9,000 years ago (Sartoretto, Verlaque, & Laborel, 1996). They are currently at a distance of 100 m to 4 km from the shore. In the north‐east and east of Marseille, several fragments of natural vegetation remain on hills poorly suited to urbanization (Lhotte, Affre, & Saatkamp, 2014). These were surrounded by urban developments—and thus separated from contiguous areas of natural habitat—between ca. 1920 and 1965 (Lhotte et al., 2014). The vegetation in the study area is characterized by a mosaic of shrubland “*garrigues”* which are dominated by *Quercus coccifera* and include open rocky spaces.

### 2.2 Study design

The study area and sampling design were intended to represent three contrasting scenarios of habitat isolation (Figure 2): (1) one large “continuous” area of natural habitat, (2) patches of natural habitat recently isolated by an urban matrix, hereafter “habitat fragments,” and (3) patches of natural habitat that have long been isolated by the Mediterranean sea, but are close to the mainland (continental‐shelf islands), hereafter “islands.”

The spatial configuration of natural habitats in the study area was obtained from the 2013 habitat map of the Parc National des Calanques. Habitat fragments outside the National Park were delimited by photo‐interpretation of ortho‐photos from 2008 (50 cm pixel size, BD Ortho, Institut Géographique National de France) and were field checked in 2015. The 1:50,000 scale regional land‐use map (Centre Régional de l'Information Géographique PACA, CRIGE PACA, 2006) was used for additional information on distance and habitat amount around the fragments.

The analyses are based on a total of 295 floristic relevés recording the presence of vascular plant species. These relevés were performed in 100 m^2^ circular plots, sampled systematically to cover natural habitats in the study area, based on a 500 × 500 m grid cell. We visited 174 plots during the period 2007–2010 (see Pouget et al., 2016), and 121 additional plots in the spring of 2015 to complete the dataset, following the same sampling design. The minimum distance between pairs of plots is 100 m, and the plots were located at a distance of 50 m from the habitat fragment border when possible.

From the total list of vascular plant species in all relevés in the study area (442 species), we removed those species that only appeared in urban habitat fragments (62 species), because we suspected that these species can also grow in the urban matrix and therefore would not be subjected to isolation processes in natural habitat fragments. All the analyses below were first conducted with all the 379 remaining species (see the list in the Supporting Materials, Table S1) and then we compared annual with perennial species only. We excluded geophytes and lianas from the perennial plants as they made very little contribution to the data and because we think they involve different patterns of mobility and vulnerability to isolation compared to perennials. Annuals differ from perennials by their more rapid life cycle, their lower space requirements, and the shorter persistence times of adult plants, potentially leading to increased rates of colonization and hence modified colonization‐extinction dynamics (*e.g.,* Lindborg, 2007).

We assumed that most of the dispersal events and relevant interactions among individuals occurred within 2‐km radius circles, and that beyond 2 km dispersal and interactions became limited. We chose 2 km as a conservative distance threshold for two reasons: (1) In our study system, homogeneity in species composition tends to decline beyond 2 km (Pouget et al., 2016). (2) Dispersal distance may vary considerably among our study species, but Vittoz and Engler (2007) found in a meta‐analysis that 99% of the seeds of zoochorous plant species—that is, those that disperse the furthest away—dispersed within 1,500 m (confirmed by Jordano, García, Godoy, & García‐Castaño, 2007).

We thus built 295 circular landscapes of 2‐km radius, centered on each floristic relevé using ArcGIS (see Supporting Materials for more details (Fig. S1)).

### 2.3 Patches as isolated ecological units

In order to test whether isolation generates further species extinctions in addition to those strictly caused by habitat loss in remnant habitat fragments, we compared empirical rates of species richness increase with increasing amount of habitat (SAR slopes) for continuous habitat, habitat fragments, and continental‐shelf islands. A recent meta‐analysis (Triantis et al., 2012) identified the power function as the best‐supported mathematical function for non‐nested SAR curves:

(1) $$S=c\cdot A^{z}\to \text{log}\left(S\right)=\text{log}\left(c\right)+z\cdot \text{log}\left(A\right),$$

with S the species richness, A the patch area or the amount of (reachable) habitat, and log(c) and *z* the intercept and slope of the log–log linear relationship, respectively. SAR curves for the different situations can be compared by comparing *z* and log(c) values.

To properly discriminate between the potential effects of *passive sampling* alone or in combination with *isolation effects*, we carefully delimited the habitat amount as an indicator of the number of interacting individuals.

In a first analysis, we considered for patchy landscapes (habitat fragments and islands) that each habitat patch delimited an isolated community, and we built SAR curves based on patch areas for 12 islands and 19 habitat fragments. We selected these 31 patches, and their surrounding circular landscapes, on the basis of the following criteria: (1) a sufficient sampling effort by patch, as explained below (Table 1); and (2) a distance of at least 1 km between the patches' centroids in order to minimize the overlap between their circular landscapes. In the continuous habitat, we obtained 24 circular landscapes after a selection based on the following criteria: (1) a distance of at least 1 km between their centroids; and (2) the centroids should be at least 500 m distant from the edge of the continuous habitat, in order to avoid landscapes not fully covered with habitat.

**Table 1:** Proportional sampling scheme to control for sampling effort (number of relevés) when habitat area (A) increases. Habitat area has been binned into three area classes

Area classes	Habitat area (A in ha)	ln(A)	Sampling effort (number of relevés)
1	0.8–6	9–11	1
2	6–44	11–13	3
3	44–327	13–15	10

To be able to compare SAR curves in all three situations (continuous, fragment, and islands), and given that (1) for islands and habitat fragments, the observed patch area ranged from 1 to 300 ha, (2) for continuous habitat, the amount of habitat within circular landscapes was always 1,256 ha (entire 2‐km radius disk covered with habitat), each hosting between 10 and 40 relevés, and that (3) species richness increases with sampling effort in a nonlinear way (Gotelli & Colwell, 2001), we needed to control for both habitat area and the corresponding sampling effort (i.e., number of relevés used to estimate species richness) within a given circular landscape (Azovsky, 2011; Cam, Nichols, Hines, Sauer, & Flather, 2002). We thus (1) binned habitat area (in patchy landscapes) into three classes, (2) used a proportional sampling scheme, that is, we subsampled more relevés for higher amounts of habitat (Borges, Hortal, Gabriel, & Homem, 2009; Schoereder & Galbiati, 2004).

In our study, the natural logarithm of fragments and islands area, log(A), ranges from 9 to 15. In order to obtain groups of comparable size, we binned this range into three classes: log(A) <11, 11 < log(A) >13, log(A) >13. For the smallest class, the sampling effort generally did not exceed one available relevé. We thus chose one relevé as a basis to estimate species richness for this class (Table 1). Based on the literature, on average, *z* = 0.355 ± 0.015 (equation (1)) (Triantis et al., 2012). This means that increasing log(A) by 2 units to move from one area class to the next largest one corresponds to doubling species richness (S multiplied by exp(2)^0.355^ ~ 2 in equation (1)). We estimated empirically the number of relevés needed to double species richness, by building rarefaction curves for each circular landscape (Gotelli & Colwell, 2001) with the library iNEXT in the R software (Hsieh, Ma, & Chao, 2016). From these rarefaction curves, we deduced that three relevés were necessary to get on average twice as many species as in one relevé (for the second area‐class), and ten relevés were necessary to double species richness again (for the largest area class, Table 1).

Species richness was then calculated from all the relevés falling within a patch (islands and fragments) by rarefying them to one, three, or ten relevés, depending on their area class (100 runs). For continuous habitat, species richness was rarefied within each circular landscape to one, three, and ten relevés successively, and corresponding rarefied richness was associated with a habitat amount equal to the mid‐point of the area class, that is, log(A) = 10, 12 or 14.

Rarefied richness and area were compared with a linear regression model, and slopes and intercepts were compared between the three situations with *t*‐tests.

### 2.4 Graph approach: the role of functional connectivity

In a second analysis, we wanted to assess the influence of functional connectivity on species richness patterns in islands and fragments. We used the approach suggested by Fahrig (2013) that maintains the sampling effort invariable while varying the amount of reachable habitat. To do so, we focused on the 12 islands and 19 habitat fragments analyzed in the previous section. We calculated three different proxies for the total number of interacting individuals according to different levels of potential dispersal. We tested ten different dispersal distances ranging from 100 to 1,000 m (Vittoz & Engler, 2007); for simplicity, the same dispersal distance was attributed to all species in each scenario.

**Figure 3:**
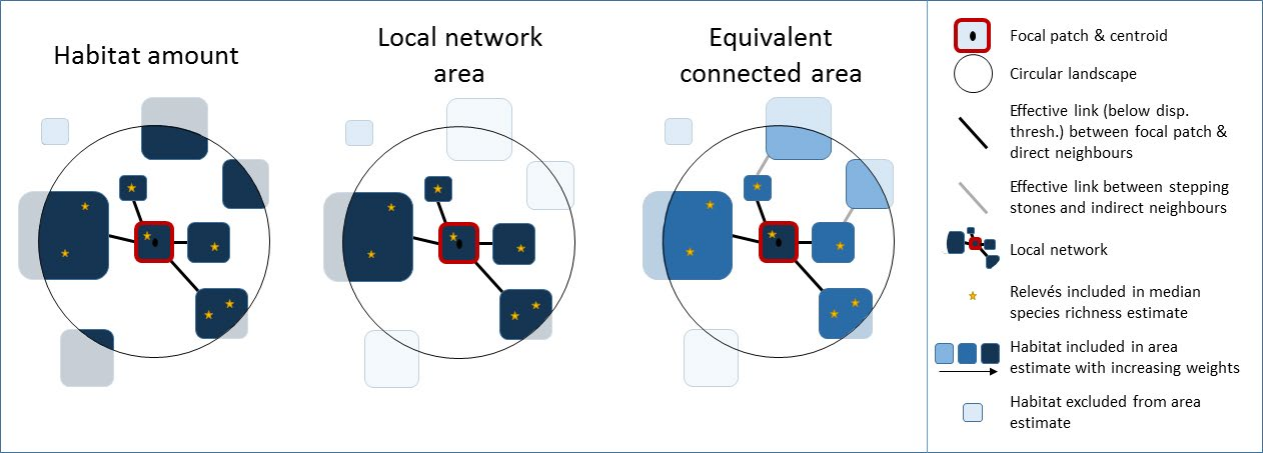
Definition of concepts and metrics used to assess functional connectivity

Patches were considered as “directly connected” to the focal patch if the distance between them was lower than the given dispersal distance (Figure 3). Within the 2‐km radius circular landscape, the focal patch and its direct neighbors formed the “local network” (Figure 3). Other patches within the circular 2‐km radius landscape could also be indirectly connected to the focal patch through their connections with direct neighbors that act as stepping stones (Figure 3). For the habitat patches that extend beyond the limits of the circular landscape, only the part falling within the circular landscape was considered relevant for the local ecological dynamics, even when they were continental (Figures 3 and S1). For each local network, species richness was estimated as the median species richness of all the relevés falling within its patches (hereafter “plot species richness,” Figure 3).

In order to explain plot species richness, we calculated three metrics assumed to be alternative ways to reflect the total number of interacting individuals.

First, we calculated the *habitat amount*,* that is,* the cumulated area of all the habitat patches within a circular landscape (Figure 3). Considering the habitat amount as a proxy for the number of interacting individuals means assuming (1) that dispersal is unlimited within 2 km around the focal patch's centroid, and (2) that the spatial arrangement of habitat areas within the circular landscape does not matter.

Second, we calculated the *local network area*,* that is,* the cumulated area of all the habitat patches within a local network (focal patch and directly connected patches, Figure 3). This variable is expected to represent the number of interacting individuals when dispersal is restricted within a given distance threshold among patches (100–1,000 m) and within 2 km around the focal patch's centroid. Here, the spatial arrangement of habitat areas within the circular landscape does matter, but the potential contribution of indirect neighbors is not accounted for.

Third, we calculated the *equivalent connected area* (hereafter ECA, in area units), a measure that reflects the degree of habitat reachability—that is, amount and connectivity—from the focal patch within the circular landscape (Saura, Estreguil, Mouton, & Rodríguez‐Freire, 2011). Here, individuals in the focal patch may interact with individuals from any other connected patches within the circular landscape (even through stepping stones), but the probability of interaction depends on the size and topology of all patches within a 2‐km radius (Figure 3):

(2) $$\text{ECA}=\sqrt{\sum_{i}\sum_{j}\frac{a_{i}a_{j}}{1+n_{ij}}}$$

where *a*_*i*/*j*_ is the area of patch *i* or *j* and *n*_*ij*_ is the number of links in the shortest path (topological distance) between patches *i* and *j*.

All indices were calculated for the ten dispersal distances using ArcGIS 10.2.2 and Conefor Sensinode software (Saura & Torné, 2009, http://www.conefor.org).

The log–log relationship between each of the calculated indicators and plot species richness was estimated using univariate regression models for the two cases separately (islands and fragments). In the absence of an isolation effect, and when sampling effort is fixed to one plot, species richness should increase with the amount of habitat in the surrounding landscape, independently of the spatial configuration of this habitat (Fahrig, 2013). In our case, a positive relationship between plot species richness and the *habitat amount* would indicate no isolation effect; no relationship between plot species richness and any of the three metrics would indicate an isolation of the habitat patches; a positive relationship between richness and the *local network area* or the *equivalent connected area* would mean that the habitat configuration matters either at the scale of the local network (direct neighbors only) or at the scale of the landscape (indirect neighbors also influence local richness).

## 3 RESULTS

### 3.1 Islands versus fragments: two different scenarios of isolation

**Figure 4:**
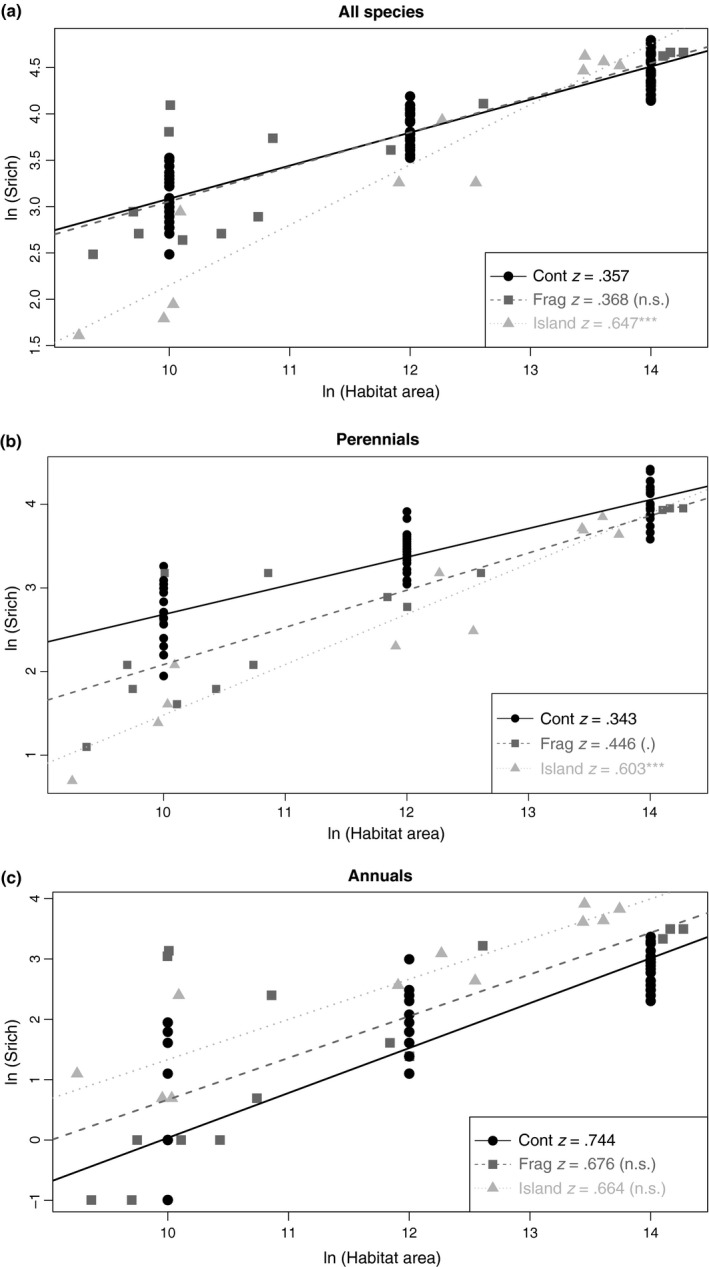
Species area curve (SAR) log‐log transformed for patches as isolated communities scenario: continuous habitat, habitat fragments, and continental‐shelf islands. The log–log *z* slope is provided, with its statistical significance for fragments and islands' parameters compared to the continuous *z* parameter: ***p <* *.001, **p <* *.01, *p <* *.05, (.) *p *<* *.1, n.s. for non‐significant. (a) All species, (b) perennial species, (c) annual species

As expected, the SAR log–log slope *z* was significantly steeper for islands than for continuous habitat when considering all species together (Figure 4a, Table S2). The slope was also steeper in islands than in continuous habitat when considering only perennial species (Figure 4b, Table S2), but was not significantly different for annual species alone (Figure 4c, Table S2). Similarly, the intercept was significantly lower for islands than for continuous habitat for all species and for perennials only.

The SAR log–log slope *z* for habitat fragments was significantly less steep than the slope for islands when considering all species or perennials only (Table S2), but was not significantly steeper than the slope for continuous habitat (Figure 4, Table S2). However, in the case of perennial species, *z* tended to be higher for habitat fragments than for continuous habitat (*p*‐value = .067 < 0.1, Table S2). In addition, the SAR intercept log(c) for perennials was also significantly lower in habitat fragments than in continuous habitat and significantly higher in habitat fragments than in islands (Table S2). Overall, for perennials, the SAR curve for habitat fragments lay between the SAR curves for continuous habitat and continental‐shelf islands.

### 3.2 The role of functional connectivity

When dispersal distance increases, the local network encompasses more patches that become directly connected to the focal patch, and thus, the pool of relevés included in the median richness estimate becomes larger.

In the case of islands, plot species richness increased significantly with the *local network area* for dispersal distances below 400 m for all species, below 700 m for perennial plants, and 200 m for annual plants (Table 2). The strongest relationships (larger slope estimates and larger *R* ^2^ values) were found for the smallest dispersal distance in each case. There was no relationship either for larger dispersal distances with the *local network area*, or for any dispersal distance for the other metrics (habitat amount and equivalent connected area). Surprisingly for annuals, plot species richness declined with increasing *local network area* when dispersal distance was above 400 m; this corresponds to a distance beyond which some continental areas might be included in the local networks centered on islands; given that islands are on average richer in annuals than continental areas (Figure 4), including plots from the continent could artificially lead to a decrease in the median plot richness for larger amounts of habitat.

**Table 2:** In islands, slope parameters and *R* ^2^ of the regressions between plot species richness and Habitat amount, Local network size, and Equivalent connected area, respectively. Slopes significantly different from 0 are in bold (*p*‐value < .05) and in italics (*p*‐value < .1)

	Dispersal capacity (meters)	100	200	300	400	500	600	700	800	900	1,000
All species											
Habitat amount	Slope	0.09	−0.26	−0.27	0.05	−0.33	−0.29	−0.33	*−0.39*	−0.35	*−0.40*
	*p*‐value	.88	.58	.56	.89	.27	.16	.16	*.08*	.11	*.08*
	*R* ^2^ adj	−.10	−.06	−.06	−.10	.03	.11	.10	*.20*	.16	*.21*
Local network size	Slope	**0.27**	**0.23**	**0.22**	0.09	*0.23*	0.11	−0.06	−0.22	−0.19	−0.23
	*p*‐value	**.00**	**.01**	**.01**	.35	*.09*	.26	.67	.21	.25	.20
	*R* ^2^ adj	**.61**	**.45**	**.44**	.00	*.19*	.04	−.08	.07	.05	.08
Equivalent connected area	Slope	0.13	−0.12	−0.13	0.10	−0.26	−0.25	−0.33	*−0.38*	−0.34	*−0.39*
	*p*‐value	.81	.79	.78	.77	.40	.22	.17	*.09*	.11	*.08*
	*R* ^2^ adj	−.09	−.09	−.09	−.09	−.02	.06	.10	*.19*	.16	*.20*
Perennials											
Habitat amount	Slope	0.24	0.18	0.05	0.21	−0.05	0.02	−0.13	−0.02	0.01	−0.06
	*p*‐value	.66	.72	.93	.42	.88	.94	.54	.93	.96	.81
	*R* ^2^ adj	−.08	−.08	−.10	−.03	−.10	−.10	−.06	−.10	−.10	−.09
Local network size	Slope	**0.26**	**0.25**	**0.26**	0.12	**0.28**	*0.25*	0.02	0.03	0.06	0.02
	*p*‐value	**.00**	**.01**	**.01**	.10	**.04**	*.06*	.85	.86	.75	.93
	*R* ^2^ adj	**.63**	**.51**	**.48**	.17	**.30**	*.24*	−.10	−.10	−.09	−.10
Equivalent connected area	Slope	0.31	0.27	0.18	0.28	0.06	0.13	−0.15	0.00	0.03	−0.04
	*p*‐value	.54	.56	.71	.28	.86	.68	.49	.99	.90	.87
	*R* ^2^ adj	−.06	−.06	−.08	.03	−.10	−.10	−.05	−.10	−.10	−.10
Annuals											
Habitat amount	Slope	−0.05	−0.58	−0.70	−0.39	**−0.75**	**−0.58**	−0.41	**−1.08**	**−0.89**	**−0.97**
	*p*‐value	.94	.15	.11	.36	**.03**	**.04**	.29	**.02**	**.03**	**.02**
	*R* ^2^ adj	−.10	.11	.16	−.01	**.33**	**.30**	.02	**.40**	**.34**	**.37**
Local network size	Slope	**0.23**	0.11	0.09	−0.03	−0.07	−0.05	−0.25	**−0.76**	**−0.64**	**−0.68**
	*p*‐value	**.04**	.22	.37	.79	.67	.71	.27	**.03**	**.04**	**.04**
	*R* ^2^ adj	**.30**	.06	−.01	−.09	−.08	−.08	.03	**.34**	**.30**	**.31**
Equivalent connected area	Slope	0.31	−0.49	−0.62	−0.39	**−0.75**	**−0.59**	−0.40	**−1.08**	**−0.89**	**−0.95**
	*p*‐value	.54	.21	.14	.36	**.03**	**.03**	.30	**.01**	**.03**	**.02**
	*R* ^2^ adj	−.10	.07	.12	−.01	**.33**	**.30**	.02	**.41**	**.35**	**.37**

In the case of habitat fragments, all three indicators failed to explain plot species richness for all dispersal distances when considering all species or annuals only (Table 3). Contrastingly, for perennials, plot species richness significantly increased with *habitat amount* and *equivalent connected area* for all dispersal distances. The strongest relationships (larger slope estimates and larger *R* ^2^ values) were found for the smallest dispersal distance.

**Table 3:** In habitat fragments, slope parameter and *R* ^2^ of the regressions between plot species richness and Habitat amount, Local network size and Equivalent connected area, respectively. Slopes significantly different from 0 are in bold (*p*‐value < .05) and in italics (*p*‐value < .1)

	Dispersal capacity (meters)	100	200	300	400	500	600	700	800	900	1,000
All species											
Habitat amount	Slope	0.15	0.12	0.13	0.05	0.10	0.11	0.13	0.11	0.09	0.05
	*p*‐value	.17	.26	.25	.61	.34	.31	.20	.25	.30	.54
	*R* ^2^ adj	.05	.02	.02	−.04	.00	.01	.04	.02	.01	−.03
Local network size	Slope	0.05	0.05	0.03	0.03	0.04	−0.03	−0.02	0.03	0.03	0.02
	*p*‐value	.36	.39	.63	.58	.54	.59	.72	.66	.60	.72
	*R* ^2^ adj	−.01	−.01	−.04	−.04	−.03	−.04	−.05	−.05	−.04	−.05
Equivalent Connected Area	Slope	0.12	0.09	0.10	0.03	0.08	0.08	0.11	0.09	0.08	0.04
	*p*‐value	.16	.28	.26	.69	.38	.34	.20	.23	.30	.54
	*R* ^2^ adj	.06	.01	.02	−.05	−.01	.00	.04	.03	.01	−.04
Perennials											
Habitat amount	Slope	**0.31**	**0.28**	**0.31**	*0.22*	*0.25*	**0.26**	**0.28**	**0.27**	**0.26**	**0.21**
	*p*‐value	**.02**	**.03**	**.02**	*.10*	*.06*	**.03**	**.01**	**.03**	**.03**	**.03**
	*R* ^2^ adj	**.23**	**.20**	**.22**	*.10*	*.06*	**.21**	**.31**	**.22**	**.21**	**.21**
Local network size	Slope	*0.12*	*0.12*	0.10	0.11	0.10	0.03	0.05	0.10	0.10	0.09
	*p*‐value	*.08*	*.09*	.15	.12	.19	.71	.45	.22	.19	.19
	*R* ^2^ adj	*.08*	*.11*	.07	.08	.04	−.05	−.02	.04	.04	.04
Equivalent connected area	Slope	**0.23**	**0.21**	**0.24**	0.17	*0.20*	**0.21**	**0.24**	**0.23**	**0.23**	**0.19**
	*p*‐value	**.02**	**.04**	**.03**	.12	*.07*	**.04**	**.01**	**.02**	**.03**	**.03**
	*R* ^2^ adj	**.23**	**.18**	**.21**	.08	*.14*	**.19**	**.30**	**.22**	**.21**	**.21**
Annuals											
Land. area	Slope	0.05	0.17	−0.02	−0.19	0.23	0.26	0.11	−0.03	−0.07	0.07
	*p*‐value	.88	.60	.96	.61	.47	.45	.77	.92	.84	.79
	*R* ^2^ adj	−.06	−.04	−.06	−.04	−.03	−.02	−.05	−.06	−.06	−.05
Local network size	Slope	−0.07	0.09	−0.07	−0.06	−0.05	−0.20	−0.12	−0.08	−0.06	−0.06
	*p*‐value	.68	.59	.69	.77	.77	.33	.57	.69	.77	.68
	*R* ^2^ adj	−.05	.59	−.05	−.05	−.05	.00	−.04	−.05	−.05	−.05
Equivalent connected area	Slope	0.02	0.11	−0.06	−0.20	0.16	0.20	0.07	−0.04	−0.08	0.06
	*p*‐value	.95	.67	.85	.49	.54	.48	.82	.90	.78	.79
	*R* ^2^ adj	−.06	−.05	−.06	−.03	−.04	−.03	−.06	−.06	−.05	−.05

## 4 DISCUSSION

This study tests how the increase in species richness with increasing habitat area differs between continuous habitat, remnant habitat fragments embedded in an urban matrix and continental‐shelf islands, in order to better understand whether the processes related to habitat spatial isolation generate further species extinctions in habitat fragments in addition to those strictly caused by habitat loss. We build on recent methodological and conceptual developments around this question to make two robust tests of these differences, based on a large dataset on systematic vegetation relevés in Mediterranean France, while controlling for sampling effort and using functional connectivity concepts to measure the amount of reachable habitat from a focal habitat patch (Fahrig, 2013; Laurance, 2008; Matthews, 2015; Mendenhall et al., 2014). Our results show that (1) species area relationships (SARs) have different slopes depending on the spatial configuration of the habitat, (2) the habitat amount hypothesis is verified in our case study, but the definition of the neighborhood matters, (3) patterns differ among annual and perennial species. Our results shed light on several major issues that are worth further discussion.

### 4.1 Are habitat fragments similar to islands?

Previous studies have predicted that in the absence of *isolation effects*, the slope of SARs should be independent of habitat spatial configuration, and should thus be the same for continuous habitat, habitat fragments, and islands. In contrast, the SAR slope reflecting an effect of isolation should be steeper than that generated by *passive sampling* alone (Fahrig, 2013; Halley et al., 2014; Watling & Donnelly, 2006). As expected, we found steeper slopes and smaller intercepts for log–log species area relationships in the case of islands than in the case of continuous habitat, suggesting that species assemblages in islands are influenced by processes that are related to their spatial isolation from other habitat areas (Hubbell, 2001; MacArthur & Wilson, 1967; Shaffer, 1981). We also found that these parameters differed little between habitat fragments and continuous habitat when considering total plant species richness. Interestingly, for perennial species, fragments showed intermediate values in SAR slope and intercept in comparison with continuous habitats and islands. This result was, however, only a trend, as slopes for habitat fragments and continuous habitat significantly differ at 0.1 only, but it has to be noted than in comparison with previous studies we here properly controlled for any bias resulting from sampling efforts that may persist when total species pools are considered in islands or fragments, whatever the sampled area or number of relevés (e.g., Matthews, Guilhaumon, Triantis, Borregaard, & Whittaker, 2016).

Such an intermediate pattern may be explained by three different hypotheses: (1) islands, habitat fragments and continuous habitat differ because they undergo contrasted environmental conditions, (2) isolation effects are lower in the case of habitat fragments than in the case of islands due to a surrounding matrix that remains partially permeable, (3) fragmentation by urban sprawl is too recent, so that processes related to habitat isolation are still ongoing and thus difficult to detect.

Firstly, one might expect that the observed differences between habitat fragments, islands, and continuous habitat may result from strong contrasts in their environmental conditions. However, there are several arguments against this explanation: (1) All our vegetation relevés have been made within a relatively small region, with rather homogenous climatic conditions, (2) we have considered near‐shore islands that are of continental origin, and despite differences in the species composition of islands and continental relevés, they are expected to host the same species pool at evolutionary scale and there have been no speciation events since the formation of the island (Pouget et al., 2013; Véla, Pavon, Giraud, Destefano, & Saatkamp, 2001), (3) all SARs are converging, meaning that for higher patch areas, species richness is no different for islands or fragments than for continuous habitat.

Secondly, one might expect that an intermediate SAR for habitat fragments by comparison with islands and continuous habitat may result from an intermediate level of isolation, similarly to what has been shown for sets of islands that differ in their levels of isolation (Triantis et al., 2012). This would mean that the urban matrix is not completely inhospitable to the taxa considered and remains partially permeable, as previously predicted for continental fragments resulting from land‐use change (Koh & Ghazoul, 2010). The matrix surrounding the habitat fragments in our study area is mainly composed of urban areas with small scattered agricultural areas (Figure 2). Our results thus support for perennial plants the findings by Matthews (2015) that the decline in species number with the decline in natural habitat area is more marked when patches are separated by an urban matrix, compared to other human land uses, but not as high as in a marine water matrix. The matrix surrounding habitat patches may clearly influence dispersal events and immigration rates among patches, but it could also have a direct negative effect on communities within patches by leading to reduced habitat quality (Saura, Martín‐Queller, & Hunter, 2014) and increased edge effects (Gonzalez et al., 2010; Laurance et al., 2006). For instance, the proximity to urban areas could be linked to higher levels of human frequentation for recreational purposes (Sanderson et al., 2002). This influence of the matrix may have stronger effects in smaller patches, due to the increased proximity of any place in the patch to the matrix, which might thus lead to similar SAR patterns.

Thirdly, in contrast with continental‐shelf islands, that were separated from the continent several thousand years ago, habitat fragments were separated from continuous habitat only decades ago. It is well‐known that it may take time before the effects of reduced immigration rates and increased extinction rates actually alter species assemblages after a fragment has been separated from continuous habitat. This phenomenon is called the “extinction debt” (Diamond, 1972; Halley & Iwasa, 2011) and means that species assemblages in habitat fragments may have not yet reached their dynamic equilibrium. This might explain why differences between SARs for habitat fragments and continuous habitat are still moderate, and this explanation would mean that the slope of the SAR for habitat fragments should increase in the future.

### 4.2 How to define the relevant neighborhood for a focal relevé?

Fahrig (2013) suggested that the relative effects of *passive sampling* and *isolation* could be further explored by investigating the relationship between species richness—measured in the focal patch under a constant sampling effort—and habitat amount in the neighborhood. In the absence of an isolation effect, species richness increases with an increasing amount of habitat in the surrounding landscape, independently of its spatial configuration.

While keeping the sampling effort constant (1 relevé), we found that total species richness in islands was positively related to the amount of habitat in the neighborhood (*local network area*) when dispersal distance was kept below 500 m. However, the slope of the relationship was at a maximum for the smallest dispersal distance (100 m), and there was no relationship with the *habitat amount* or *equivalent connected area*, suggesting that complete isolation may be an acceptable assumption to understand local species richness patterns in islands. Species richness in islands is thus mainly related to patch area, as predicted by the Theory of Island Biogeography (MacArthur & Wilson, 1967), and to a much lower extent to the size and distance of neighboring islands; the neighborhood effect results from close patches connected through direct dispersal.

For habitat fragments, we found no relationship between total plot species richness and the different variables reflecting the amount of habitat in the surrounding landscape, also suggesting full isolation. The same absence of pattern was obtained for annual species. Contrastingly, for perennials, we found a significant relationship between richness and *habitat amount* and *equivalent connected area*. Given that these relationships hold for the whole range of dispersal distances, and that slopes and *R* ^2^ were slightly higher in the case of *habitat amount* (when topology is not accounted for) than in the case of *equivalent connected area* (which accounted for the degree of interactions between the individuals in the pool), solely the amount of habitat in the surrounding landscape seems to matter, independently of its spatial configuration. This result is in line with the “habitat amount hypothesis,” recently postulated by Fahrig (2013), which predicts that species richness in a sample site does not depend only on the area of the habitat patch, but more broadly on the amount of habitat area in the surrounding landscape (also see Seibold et al., 2017).

Thus, in agreement with the first analysis, the results from the second analysis confirm that fragments are not islands and that perennials and annuals differ in how they respond to isolation effects. In addition, this second analysis shows that isolation is a question of neighborhood scale. While we have shown that small habitat fragments tend to have less rich species pools (perennials) than equivalent portions of continuous habitat, it seems that increasing the (reachable) amount of habitat in their neighborhood effectively increases this richness (Akatov, Chefranov, & Akatova, 2005; Pärtel & Zobel, 1999; Schamp, Laird, & Aarssen, 2002). This seems consistent with the hypothesis that the matrix surrounding habitat fragments is more permeable than water, especially for perennial plants (e.g., Butaye, Jacquemyn, Honnay, & Hermy, 2002).

Two main limits can be highlighted here regarding the slight effect of spatial configuration that we found. Firstly, our dataset contains a limited number of replicates for each isolation type (fragments vs. islands), and they do not represent a clear semi‐experimental design with fully uncorrelated habitat amount and spatial configuration, as suggested by Pasher et al. (2013), thus potentially overriding the potential differences between these variables (but see Haddad et al., 2016). Secondly, there is a clear limit to using unique dispersal distances for pools of diverse species (Vittoz & Engler, 2007), and a division of species into more refined functional groups could help to better disentangle their responses to habitat area and isolation, but the data requirements for doing so would be huge and the definition of the pool of interacting individuals would become overcomplicated.

### 4.3 Annuals and perennials respond differently to habitat amount and isolation

In our analysis, richness of perennial plants significantly increased with *habitat amount* and *equivalent connected area*, contrasting with no effects for annuals. Perennials also showed intermediate SAR slopes for fragments compared for islands and continuous habitats, where annuals showed no isolation effects.

Compared to perennials, annual plants are smaller in size, they need less space to establish individuals and populations and have a greater number of individuals within the same area; they also produce more seeds and they realize their life cycle and dispersal in shorter time (Moles, Falster, Leishman, & Westoby, 2004), and are less dependent on pollinators for effective reproduction (Aarssen, 2000) compared to perennials (Lhotte et al., 2014). Perennials have a much longer life span and later reproductive age, as well as larger seeds and shorter‐lived soil seed banks (Grime, Hodgson, & Hunt, 1988). These differences imply different spatial and temporal scales of interaction between a population's dynamics and spatial landscape characteristics, thus leading to four major differences regarding their ability to persist in small and isolated habitat patches: (1) In small patches, annuals can thrive due to still large populations, even in urban areas (Dornier, Pons, & Cheptou, 2011); whereas perennials have critically limited populations and decline; (2) When annual plant richness can easily be restored from the soil seed bank after severe disturbances (von Blanckenhagen & Poschlod, 2005; Bossuyt & Honnay, 2008), perennials would more easily become extinct, due to smaller or inexistent seed banks; (3) Individual perennial plants might survive over long periods, even if they no longer belong to a functional population (Johansson, Cousins, & Eriksson, 2011); (4) Moreover, the shorter life cycle of annuals may allow longer dispersal distances by the progressive migration to new patches, in stepping stone fashion (Saura, Bodin, & Fortin, 2014). Altogether, this explains how annuals can maintain high diversity in landscapes where fragmentation (patch size and isolation) processes may more easily affect populations of perennial plants.

At the scales studied here, annual plants thus seem to be less vulnerable to habitat fragmentation than perennials, which need a greater amount of habitat and higher levels of connectivity to persist in the landscape. Both SAR and neighborhood analysis suggested a contrasted response of annuals compared to perennial plants. We therefore advocate the use of consistently narrower functional groups, based on trait information such as life cycles and dispersal capacity (Matthews, Cottee‐Jones, & Whittaker, 2014).

## 5 CONCLUSION

Our study builds on recent methodological and conceptual developments, including a strict control for sampling effort, to test whether processes related to habitat spatial isolation generate further species extinctions in habitat fragments in addition to those strictly caused by habitat loss. Our results show that habitat fragments present intermediate relationships between species richness and patch area in comparison with those obtained for islands and continuous habitat. This most likely results from an intermediate level of permeability in the matrix surrounding fragments, as compared to habitat or water or from an ongoing extinction debt. Moreover, in contrast with islands, in which the species pool is mainly driven by island area, species richness in habitat fragments is related to the amount of habitat in the surrounding landscape, independently of configuration. This suggests that lower richness in smaller fragments for perennial plants is a result of intermediate levels of isolation and that extinction debt could be smaller than expected due to still ongoing dynamics of colonization‐extinction resulting from a semi‐permeable matrix. This is also consistent with the habitat amount hypothesis, namely that what matters in terms of local species pool is the amount of surrounding habitat and not its spatial arrangement. Finally, different patterns among annual and perennial species call for a greater attention to functional differences in plants, especially larger long‐lived species may be exposed to increased risks of extinction in fragmented landscapes, as compared to small short‐lived ones. Overall, our findings highlight the interest of conservation or restoration of a sufficient amount of natural habitat in the landscape and on increasing matrix permeability, in order to mitigate effects of fragmentation on local perennial plant species richness.

## CONFLICT OF INTEREST

None declared.
