# Supplementary material for: Islands, mainland, and terrestrial fragments: How isolation shapes plant diversity
Source: Ecol Evol. 2017 Jul 28;7(17):6904–17. doi: 10.1002/ece3.3150 (PMC5587450; doi:10.1002/ece3.3150)
Supplement: Supplementary file 1 [file ECE3-7-6904-s001.docx]

**SUPPORTING INFORMATION**

**Figure S1 - Patch extent within circular landscape** - Only the habitat areas (grey) falling within circular landscapes (dark grey) were considered as relevant for the local ecological dynamics that shape species assemblages in the sampled relevés (white dots), and then accounted for in the metrics calculation.


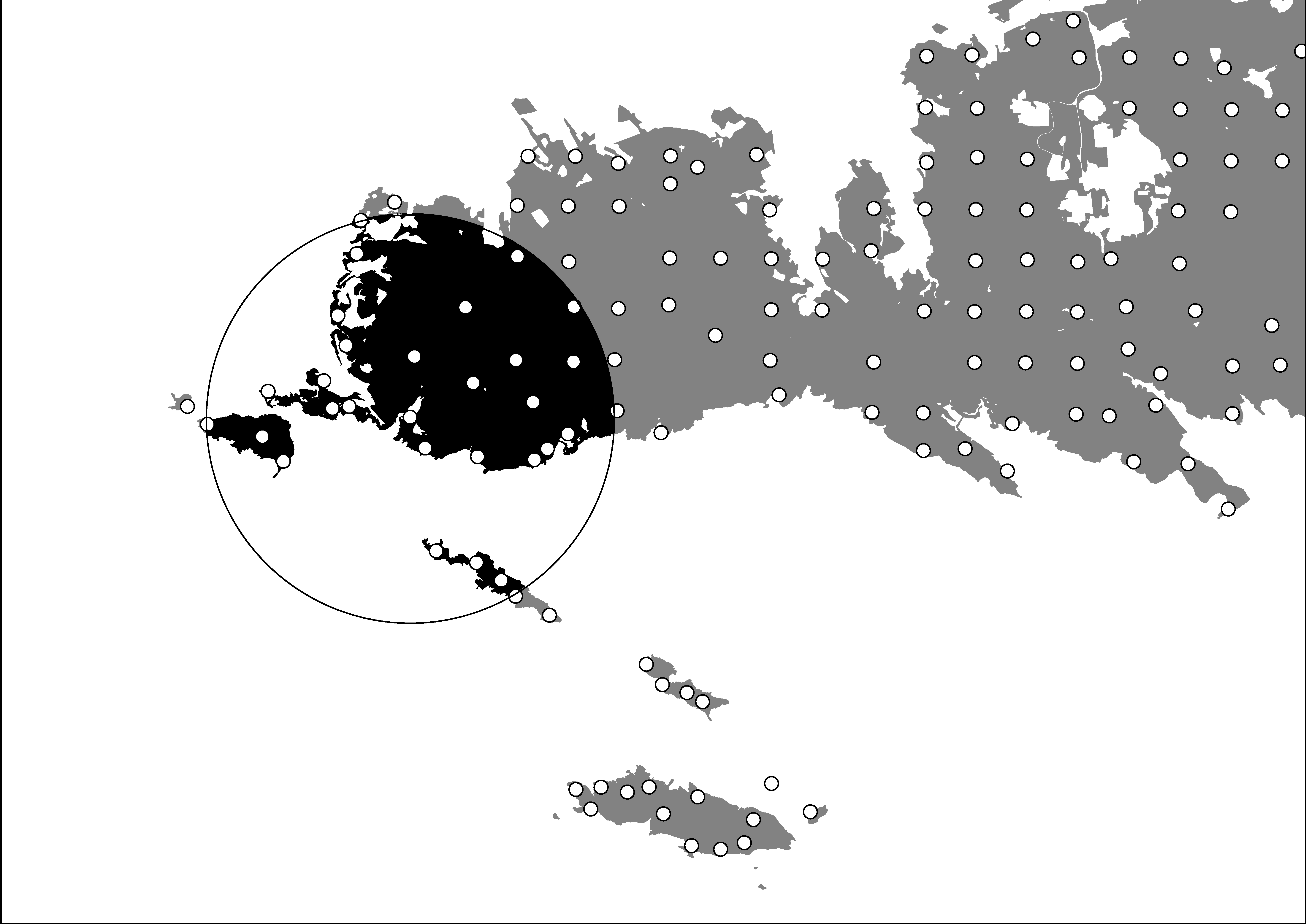

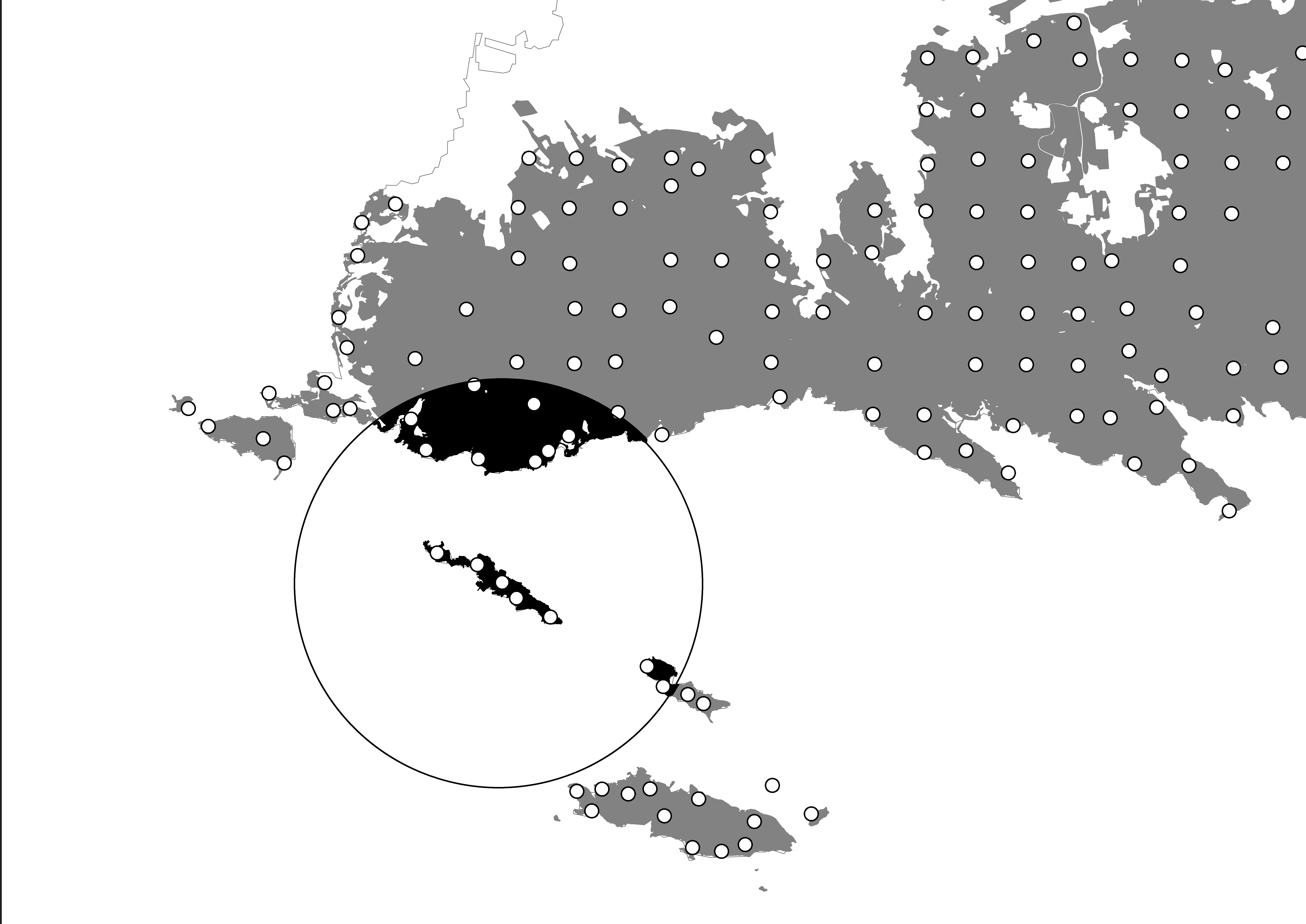


**Table S1.** List of the 279 plant species of the study, their Raunkiaer type, P: Phanerophytes, NP: Nanophanerophytes, C: Chamephytes, H: Hemicryptophytes, G: Geophytes, PL: Climbing plants, T: Therophytes; and the two groups of life forms used in this study: Perennials and annuals.

| Scientific name | Raunkiaer | Life form |
| --- | --- | --- |
| *Achnaterum bromoides* | H | PERENNIAL |
| *Aegilops ovata* | T | ANNUAL |
| *Aetheorhiza bulbosa* | G |  |
| *Agave americana* | NP | PERENNIAL |
| *Allium acutiflorum* | G |  |
| *Allium commutatum* | G |  |
| *Allium porrum* | G |  |
| *Allium sphaerocephalon* | G |  |
| *Amelanchier ovalis* | NP | PERENNIAL |
| *Anagallis arvensis* | T | ANNUAL |
| *Andryala integrifolia* | T | ANNUAL |
| *Anemone palmata* | G |  |
| *Anthericum liliago* | G |  |
| *Anthemis secundiramea* | T | ANNUAL |
| *Anthyllis vulneraria* | H | PERENNIAL |
| *Antirrhinum latifolium* | H | PERENNIAL |
| *Aphyllanthes monspeliensis* | C | PERENNIAL |
| *Arabis auriculata* | T | ANNUAL |
| *Arabis collina* | H | PERENNIAL |
| *Arabis hirsuta* | H | PERENNIAL |
| *Arbutus unedo* | P | PERENNIAL |
| *Arenaria aggregata* | C | PERENNIAL |
| *Arenaria modesta* | T | ANNUAL |
| *Arenaria serpyllifolia* | T | ANNUAL |
| *Argyrolobium zanonii* | C | PERENNIAL |
| *Aristolochia pistolochia* | G |  |
| *Arisarum vulgare* | G |  |
| *Armeria arenaria subsp. bupleuroides* | H | PERENNIAL |
| *Arrhenatherum elatius* | H | PERENNIAL |
| *Artemisia caerulescens subsp. gallica* | C | PERENNIAL |
| *Arthrocnemum macrostachyum* | C | PERENNIAL |
| *Asparagus acutifolius* | PL |  |
| *Asperula cynanchica* | H | PERENNIAL |
| *Asphodelus cerasiferus* | G |  |
| *Asphodelus fistulosus* | G |  |
| *Asphodelus ramosus* | G |  |
| *Asplenium ruta-muraria* | G |  |
| *Asplenium trichomanes* | G |  |
| *Asterolinon linum-stellatum* | T | ANNUAL |
| *Aster sedifolius* | H | PERENNIAL |
| *Astragalus tragacantha* | C | PERENNIAL |
| *Atriplex prostrata* | T | ANNUAL |
| *Avena barbata* | T | ANNUAL |
| *Avenula bromoides* | H | PERENNIAL |
| *Avena pratensis* | H | PERENNIAL |
| *Avena sterilis* | T | ANNUAL |
| *Bellis sylvestris* | H | PERENNIAL |
| *Beta vulgaris subsp. maritima* | C | PERENNIAL |
| *Biscutella laevigata* | H | PERENNIAL |
| *Bituminaria bituminosa* | H | PERENNIAL |
| *Blackstonia perfoliata* | T | ANNUAL |
| *Brachypodium distachyon* | T | ANNUAL |
| *Brachypodium retusum* | H | PERENNIAL |
| *Bromus diandrus* | T | ANNUAL |
| *Bromus erectus* | H | PERENNIAL |
| *Bromus hordeaceus* | T | ANNUAL |
| *Bromus madritensis* | T | ANNUAL |
| *Bromus rubens* | T | ANNUAL |
| *Bupleurum baldense* | T | ANNUAL |
| *Bupleurum fruticosum* | NP | PERENNIAL |
| *Calendula arvensis* | T | ANNUAL |
| *Campanula erinus* | T | ANNUAL |
| *Camphorosma monspeliaca* | C | PERENNIAL |
| *Campanula rotundifolia* | H | PERENNIAL |
| *Capsella bursa-pastoris* | H | PERENNIAL |
| *Carduus pycnocephalus* | H | PERENNIAL |
| *Carduus tenuiflorus* | H | PERENNIAL |
| *Carex distachya* | H | PERENNIAL |
| *Carex flacca* | H | PERENNIAL |
| *Carex halleriana* | H | PERENNIAL |
| *Carex humilis* | H | PERENNIAL |
| *Carlina corymbosa* | H | PERENNIAL |
| *Catapodium loliaceum* | T | ANNUAL |
| *Catapodium rigidum* | T | ANNUAL |
| *Celtis australis* | P | PERENNIAL |
| *Centaurea aspera subsp. aspera* | H | PERENNIAL |
| *Centranthus calcitrapa* | T | ANNUAL |
| *Centaurea melitensis* | T | ANNUAL |
| *Centaurea paniculata* | H | PERENNIAL |
| *Centranthus ruber* | C | PERENNIAL |
| *Cephalaria leucantha* | C | PERENNIAL |
| *Cerastium pumilum* | T | ANNUAL |
| *Cercis siliquastrum* | P | PERENNIAL |
| *Cervaria rivini* | H | PERENNIAL |
| *Ceterach officinarum* | H | PERENNIAL |
| *Cistus albidus* | NP | PERENNIAL |
| *Cistus monspeliensis* | C | PERENNIAL |
| *Cistus salviifolius* | NP | PERENNIAL |
| *Clematis flammula* | PL |  |
| *Clypeola jonthlaspi* | T | ANNUAL |
| *Convolvulus althaeoides* | H | PERENNIAL |
| *Convolvulus cantabricus* | C | PERENNIAL |
| *Conyza sumatrensis* | T | ANNUAL |
| *Coris monspeliensis* | H | PERENNIAL |
| *Coronilla juncea* | NP | PERENNIAL |
| *Coronilla minima* | C | PERENNIAL |
| *Coronilla valentina subsp. glauca* | NP | PERENNIAL |
| *Crataegus monogyna* | P | PERENNIAL |
| *Crepis albida* | H | PERENNIAL |
| *Crepis foetida* | T | ANNUAL |
| *Crepis leontodontoides* | H | PERENNIAL |
| *Crepis micrantha* | T | ANNUAL |
| *Crepis sancta* | T | ANNUAL |
| *Crepis suffreniana* | T | ANNUAL |
| *Crepis vesicaria* | H | PERENNIAL |
| *Crithmum maritimum* | C | PERENNIAL |
| *Crucianella angustifolia* | T | ANNUAL |
| *Crucianella latifolia* | T | ANNUAL |
| *Crupina vulgaris* | T | ANNUAL |
| *Cupressus arizonica* | P | PERENNIAL |
| *Cuscuta epithymum* | T | ANNUAL |
| *Cynoglossum creticum* | H | PERENNIAL |
| *Cynosurus echinatus* | T | ANNUAL |
| *Cytisophyllum sessilifolium* | NP | PERENNIAL |
| *Chaenorrhinum origanifolium* | H | PERENNIAL |
| *Chaenorrhinum rubrifolium* | T | ANNUAL |
| *Cheirolophus intybaceus* | H | PERENNIAL |
| *Chenopodium album* | T | ANNUAL |
| *Chenopodium murale* | T | ANNUAL |
| *Chondrilla juncea* | H | PERENNIAL |
| *Dactylis glomerata subsp. hispanica* | H | PERENNIAL |
| *Daphne gnidium* | NP | PERENNIAL |
| *Daucus carota* | H | PERENNIAL |
| *Daucus gingidium* | H | PERENNIAL |
| *Dianthus sylvestris* | H | PERENNIAL |
| *Diplotaxis muralis* | T | ANNUAL |
| *Diplotaxis tenuifolia* | H | PERENNIAL |
| *Dittrichia viscosa* | C | PERENNIAL |
| *Dorycnium pentaphyllum* | C | PERENNIAL |
| *Echium calycinum* | T | ANNUAL |
| *Echinops ritro* | H | PERENNIAL |
| *Echium vulgare* | H | PERENNIAL |
| *Ephedra distachya* | NP | PERENNIAL |
| *Erica multiflora* | NP | PERENNIAL |
| *Erodium ciconium* | H | PERENNIAL |
| *Erodium cicutarium* | T | ANNUAL |
| *Erodium chium* | T | ANNUAL |
| *Erodium malacoides* | T | ANNUAL |
| *Eryngium campestre* | G |  |
| *Euphorbia characias* | C | PERENNIAL |
| *Euphorbia exigua* | T | ANNUAL |
| *Euphorbia linifolia* | C | PERENNIAL |
| *Euphorbia peplus* | T | ANNUAL |
| *Euphorbia segetalis* | T | ANNUAL |
| *Euphorbia serrata* | H | PERENNIAL |
| *Euphorbia sulcata* | T | ANNUAL |
| *Festuca cinerea* | H | PERENNIAL |
| *Festuca ovina* | H | PERENNIAL |
| *Filago pyramidata* | T | ANNUAL |
| *Foeniculum vulgare* | H | PERENNIAL |
| *Frankenia hirsuta* | C | PERENNIAL |
| *Frankenia pulverulenta* | T | ANNUAL |
| *Fumana laevipes* | C | PERENNIAL |
| *Fumana laevis* | C | PERENNIAL |
| *Fumaria bicolor* | T | ANNUAL |
| *Fumaria capreolata* | T | ANNUAL |
| *Fumana ericoides* | C | PERENNIAL |
| *Fumana thymifolia* | C | PERENNIAL |
| *Galactites elegans* | H | PERENNIAL |
| *Galeopsis angustifolia* | T | ANNUAL |
| *Galium aparine* | T | ANNUAL |
| *Galium corrudifolium* | H | PERENNIAL |
| *Galium lucidum* | H | PERENNIAL |
| *Galium murale* | T | ANNUAL |
| *Galium parisiense* | T | ANNUAL |
| *Galium pumilum* | H | PERENNIAL |
| *Galium pusillum* | H | PERENNIAL |
| *Gastridium ventricosum* | T | ANNUAL |
| *Genista hispanica* | C | PERENNIAL |
| *Genista lobelii* | C | PERENNIAL |
| *Genista pilosa* | C | PERENNIAL |
| *Geranium molle* | T | ANNUAL |
| *Geranium robertianum* | H | PERENNIAL |
| *Geranium rotundifolium* | T | ANNUAL |
| *Globularia alypum* | C | PERENNIAL |
| *Gouffeia arenarioides* | T | ANNUAL |
| *Halimione portulacoides* | C | PERENNIAL |
| *Hedera helix* | PL |  |
| *Hedypnois rhagadioloides* | T | ANNUAL |
| *Helianthemum apenninum* | C | PERENNIAL |
| *Helianthemum pilosum* | C | PERENNIAL |
| *Helichrysum stoechas* | C | PERENNIAL |
| *Helianthemum syriacum* | C | PERENNIAL |
| *Helianthemum oelandicum subsp. incanum* | C | PERENNIAL |
| *Helianthemum oelandicum subsp. italicum* | C | PERENNIAL |
| *Hieracium murorum* | H | PERENNIAL |
| *Hieracium pilosella* | H | PERENNIAL |
| *Himantoglossum robertianum* | G |  |
| *Hippocrepis biflora* | T | ANNUAL |
| *Hippocrepis ciliata* | T | ANNUAL |
| *Hordeum murinum* | T | ANNUAL |
| *Hornungia petraea* | T | ANNUAL |
| *Hymenolobus procumbens subsp. revelieri* | T | ANNUAL |
| *Hyoscyamus albus* | H | PERENNIAL |
| *Hyoseris radiata* | H | PERENNIAL |
| *Hypericum perforatum* | H | PERENNIAL |
| *Hypochaeris glabra* | T | ANNUAL |
| *Iberis linifolia* | H | PERENNIAL |
| *Inula crithmoides subsp. longifolia* | C | PERENNIAL |
| *Inula montana* | H | PERENNIAL |
| *Iris lutescens* | G |  |
| *Jasminum fruticans* | NP | PERENNIAL |
| *Juniperus oxycedrus* | P | PERENNIAL |
| *Juniperus phoenicea* | P | PERENNIAL |
| *Knautia purpurea* | H | PERENNIAL |
| *Koeleria vallesiana* | H | PERENNIAL |
| *Lactuca perennis* | H | PERENNIAL |
| *Lactuca serriola* | T | ANNUAL |
| *Lactuca viminea* | H | PERENNIAL |
| *Lagurus ovatus* | T | ANNUAL |
| *Lamium amplexicaule* | T | ANNUAL |
| *Laserpitium gallicum* | H | PERENNIAL |
| *Lavatera arborea* | H | PERENNIAL |
| *Lavandula latifolia* | C | PERENNIAL |
| *Leontodon crispus* | H | PERENNIAL |
| *Leontodon tuberosus* | H | PERENNIAL |
| *Leuzea conifera* | H | PERENNIAL |
| *Limonium echioides* | T | ANNUAL |
| *Limonium pseudominutum* | C | PERENNIAL |
| *Limonium virgatum* | C | PERENNIAL |
| *Linaria simplex* | T | ANNUAL |
| *Linaria supina* | C | PERENNIAL |
| *Linum campanulatum* | C | PERENNIAL |
| *Linum narbonense* | C | PERENNIAL |
| *Linum strictum* | T | ANNUAL |
| *Linum suffruticosum* | C | PERENNIAL |
| *Lobularia maritima* | C | PERENNIAL |
| *Lonicera implexa* | PL |  |
| *Lotus cytisoides subsp. Cytisoides* | C | PERENNIAL |
| *Lotus edulis* | T | ANNUAL |
| *Lycopersicon esculentum* | T | ANNUAL |
| *Malva parviflora* | T | ANNUAL |
| *Malva sylvestris* | H | PERENNIAL |
| *Matthiola incana* | C | PERENNIAL |
| *Medicago arborea* | NP | PERENNIAL |
| *Medicago littoralis* | T | ANNUAL |
| *Medicago minima* | T | ANNUAL |
| *Medicago orbicularis* | T | ANNUAL |
| *Medicago polymorpha* | T | ANNUAL |
| *Melica amethystina* | H | PERENNIAL |
| *Melica ciliata* | H | PERENNIAL |
| *Melica minuta* | H | PERENNIAL |
| *Melilotus sp.* | T | ANNUAL |
| *Mercurialis annua* | T | ANNUAL |
| *Minuartia hybrida* | T | ANNUAL |
| *Misopates orontium* | T | ANNUAL |
| *Muscari comosum* | G |  |
| *Muscari neglectum* | G |  |
| *Narcissus dubius* | G |  |
| *Odontites luteus* | T | ANNUAL |
| *Olea europaea* | P | PERENNIAL |
| *Ononis minutissima* | C | PERENNIAL |
| *Ophrys fusca* | G |  |
| *Opuntia ficus-indica* | NP | PERENNIAL |
| *Orchis olbiensis* | G |  |
| *Ornithogalum gussonei* | G |  |
| *Osyris alba* | NP | PERENNIAL |
| *Pallenis maritima* | H | PERENNIAL |
| *Pallenis spinosa* | H | PERENNIAL |
| *Pancratium maritimum* | G |  |
| *Papaver dubium* | T | ANNUAL |
| *Papaver rhoeas* | T | ANNUAL |
| *Papaver somniferum* | T | ANNUAL |
| *Parapholis incurva* | T | ANNUAL |
| *Parietaria judaica* | H | PERENNIAL |
| *Parietaria lusitanica* | T | ANNUAL |
| *Petrorhagia prolifera* | T | ANNUAL |
| *Phagnalon sordidum* | C | PERENNIAL |
| *Phelipanche rosmarina* | T | ANNUAL |
| *Phillyrea angustifolia* | NP | PERENNIAL |
| *Phillyrea media* | NP | PERENNIAL |
| *Phillyrea latifolia* | P | PERENNIAL |
| *Picris hieracioides* | H | PERENNIAL |
| *Pimpinella tragium* | H | PERENNIAL |
| *Pinus halepensis* | P | PERENNIAL |
| *Piptatherum caerulescens* | H | PERENNIAL |
| *Piptatherum miliaceum* | H | PERENNIAL |
| *Pistacia lentiscus* | NP | PERENNIAL |
| *Pistacia terebinthus* | P | PERENNIAL |
| *Plantago lagopus* | T | ANNUAL |
| *Plantago lanceolata* | H | PERENNIAL |
| *Plantago sempervirens* | C | PERENNIAL |
| *Plantago subulata* | C | PERENNIAL |
| *Poa annua* | T | ANNUAL |
| *Poa bulbosa* | H | PERENNIAL |
| *Polygala rupestris* | C | PERENNIAL |
| *Polycarpon tetraphyllum* | T | ANNUAL |
| *Potentilla hirta* | H | PERENNIAL |
| *Ptychotis saxifraga* | H | PERENNIAL |
| *Quercus coccifera* | P | PERENNIAL |
| *Quercus ilex* | P | PERENNIAL |
| *Quercus pubescens* | P | PERENNIAL |
| *Raphanus raphanistrum* | T | ANNUAL |
| *Rapistrum rugosum* | T | ANNUAL |
| *Reichardia picroides* | H | PERENNIAL |
| *Rhagadiolus stellatus* | T | ANNUAL |
| *Rhamnus alaternus* | P | PERENNIAL |
| *Rhus coriaria* | NP | PERENNIAL |
| *Rosa canina* | NP | PERENNIAL |
| *Rosmarinus officinalis* | NP | PERENNIAL |
| *Rubia peregrina* | PL |  |
| *Rubus canescens* | NP | PERENNIAL |
| *Rubus ulmifolius* | NP | PERENNIAL |
| *Rumex intermedius* | H | PERENNIAL |
| *Ruscus aculeatus* | C | PERENNIAL |
| *Ruta angustifolia* | C | PERENNIAL |
| *Sagina apetala* | T | ANNUAL |
| *Sanguisorba minor* | H | PERENNIAL |
| *Santolina chamaecyparissus* | C | PERENNIAL |
| *Saponaria ocymoides* | H | PERENNIAL |
| *Saxifraga tridactylites* | T | ANNUAL |
| *Scandix australis* | T | ANNUAL |
| *Scilla autumnalis* | G |  |
| *Scorpiurus muricatus* | T | ANNUAL |
| *Scrophularia lucida* | H | PERENNIAL |
| *Schoenus nigricans* | H | PERENNIAL |
| *Sedum acre* | C | PERENNIAL |
| *Sedum album* | C | PERENNIAL |
| *Sedum anopetalum* | C | PERENNIAL |
| *Sedum dasyphyllum* | C | PERENNIAL |
| *Sedum litoreum subsp. Litoreum* | T | ANNUAL |
| *Sedum sediforme* | C | PERENNIAL |
| *Senecio cineraria* | C | PERENNIAL |
| *Senecio leucanthemifolius* | T | ANNUAL |
| *Senecio vulgaris* | T | ANNUAL |
| *Sesleria caerulea* | H | PERENNIAL |
| *Sherardia arvensis* | T | ANNUAL |
| *Sideritis endressii* | C | PERENNIAL |
| *Sideritis romana* | T | ANNUAL |
| *Silene italica* | H | PERENNIAL |
| *Silene nocturna* | T | ANNUAL |
| *Silene otites* | H | PERENNIAL |
| *Silene saxifraga* | H | PERENNIAL |
| *Silene sedoides* | T | ANNUAL |
| *Silene vulgaris* | H | PERENNIAL |
| *Sisymbrium irio* | T | ANNUAL |
| *Sisymbrium polyceratium* | T | ANNUAL |
| *Sixalix atropurpurea subsp. maritima* | H | PERENNIAL |
| *Smilax aspera* | PL |  |
| *Sonchus asper subsp. glaucescens* | H | PERENNIAL |
| *Sonchus asper* | T | ANNUAL |
| *Sonchus oleraceus* | T | ANNUAL |
| *Sonchus tenerrimus* | T | ANNUAL |
| *Sorbus domestica* | P | PERENNIAL |
| *Stachys brachyclada* | T | ANNUAL |
| *Staehelina dubia* | C | PERENNIAL |
| *Stellaria media* | T | ANNUAL |
| *Stipa eriocaulis* | H | PERENNIAL |
| *Stipa offneri* | H | PERENNIAL |
| *Suaeda splendens* | T | ANNUAL |
| *Suaeda vera* | NP | PERENNIAL |
| *Taraxacum obovatum* | H | PERENNIAL |
| *Teucrium aureum* | C | PERENNIAL |
| *Teucrium chamaedrys* | C | PERENNIAL |
| *Teucrium flavum* | C | PERENNIAL |
| *Teucrium montanum* | C | PERENNIAL |
| *Teucrium polium* | C | PERENNIAL |
| *Thesium divaricatum* | C | PERENNIAL |
| *Thymelaea hirsuta* | C | PERENNIAL |
| *Thymelaea tartonraira subsp. tartonraira* | NP | PERENNIAL |
| *Thymus vulgaris* | C | PERENNIAL |
| *Torilis arvensis* | T | ANNUAL |
| *Tragopogon porrifolius subsp. australis* | T | ANNUAL |
| *Trifolium scabrum* | T | ANNUAL |
| *Trinia glauca* | H | PERENNIAL |
| *Tulipa sylvestris subsp. australis* | G |  |
| *Tyrimnus leucographus* | H | PERENNIAL |
| *Ulex parviflorus* | C | PERENNIAL |
| *Urospermum dalechampii* | H | PERENNIAL |
| *Urospermum picroides* | T | ANNUAL |
| *Urtica urens* | T | ANNUAL |
| *Valantia muralis* | T | ANNUAL |
| *Veronica arvensis* | T | ANNUAL |
| *Viburnum tinus* | P | PERENNIAL |
| *Vicia sativa* | T | ANNUAL |
| *Vincetoxicum hirundinaria* | H | PERENNIAL |
| *Viola alba* | H | PERENNIAL |
| *Viola odorata* | H | PERENNIAL |
| *Vulpia ciliata* | T | ANNUAL |

**Table S2.** Regression parameters, intercept log(c) and z log-log slope, for the three isolation scenarios, and the statistical difference (p-value) of parameters in continental-shelf islands, as compared to continuous habitat (‘cont’), and in habitat fragments, as compared to islands and to continuous habitat. Different groups of species were analysed: all species, perennials only and annuals only. P-values below 0.05 are indicated in bold and those below 0.1 in italics.

|  |  | **All species** | **Perennial species** | **Annual species** |
| --- | --- | --- | --- | --- |
| **Continuous** | **log(c)** | -0.481 | -0.745 | -7.407 |
|  | **p-value (Different from 0)** | *0.068* | **0.012** | **<2e-16** |
|  | **z** | 0.357 | 0.343 | 0.744 |
|  | **p-value (Different from 0)** | **< 2e-16** | **< 2e-16** | **< 2e-16** |
| **Islands** | **log(c)** | -4.349 | -4.552 | -6.250 |
|  | **p-value (Difference with cont. c parameter)** | **0.000** | **0.000** | 0.467 |
|  | **z** | 0.647 | 0.603 | 0.664 |
|  | **p-value (Difference with cont. z parameter)** | **0.000** | **0.000** | 0.596 |
| **Habitat fragments** | **log(c)** | -0.672 | -2.364 | -7.486 |
|  | **p-value (Difference with cont. c parameter)** | 0.739 | **0.013** | 0.609 |
|  | **p-value (Difference with island. c parameter)** | **0.000** | **0.016** | 0.677 |
|  | **z** | 0.368 | 0.446 | 0.676 |
|  | **p-value (Difference with cont. z parameter)** | 0.817 | *0.063* | 0.619 |
|  | **p-value (Difference with island. z parameter)** | **0.000** | **0.037** | 0.890 |
